# Digital workflow feasibility for the fabrication of intraoral maxillofacial prosthetics after surgical resection: a systematic literature review

**DOI:** 10.2340/aos.v83.40870

**Published:** 2024-06-19

**Authors:** Gunjan Srivastava, Subrat Kumar Padhiary, Neeta Mohanty, Pravinkumar G. Patil, Saurav Panda, Carlos Cobo-Vazquez, Gülce Çakmak, Pedro Molinero-Mourelle

**Affiliations:** a Department of Prosthodontics, Institute of Dental Sciences, Siksha ‘O’ Anusandhan Deemed to be University, Bhubaneswar, India; b Department of Oral and Maxillofacial Surgery, Institute of Dental Sciences, Siksha ‘O’ Anusandhan Deemed to be Univesity, Bhubaneswar, India; c Department of Oral and Maxillofacial Pathology, Institute of Dental Sciences, Siksha ‘O’ Anusandhan Deemed to be University, Bhubaneswar, India; d Department of Prosthodontics, Division of Restorative Dentistry, School of Dentistry, International Medical University, Kuala Lumpur, Malaysia; e Department of Periodontics, Institute of Dental Sciences, Siksha ‘O’ Anusandhan, University, Bhubaneswar, India; f Department of Dental Clinical Specialties, Faculty of Dentistry, Complutense University of Madrid, Madrid, Spain; g Department of Reconstructive Dentistry and Gerodontology, School of Dental Medicine, University of Bern, Bern, Switzerland

**Keywords:** Maxillofacial prosthetics, digital workflow, intraoral scanning, CBCT, CAD-CAM

## Abstract

**Objectives:**

To evaluate the current evidence of digital workflow feasibility based on the data acquisition methods and the software tools used to fabricate intraoral prostheses for patients with partial or total maxillary and mandibular defects.

**Materials and methods:**

An electronic search was performed in PubMed, SCOPUS, and Web of Science using a combination of relevant keywords: digital workflow, digital designing, computer-assisted design-computer aided manufacturing, 3D printing, maxillectomy, and mandibulectomy. The Joanna Briggs Institute Critical Appraisal Tool was used to assess the quality of evidence in the studies reviewed.

**Results:**

From a total of 542 references, 33 articles were selected, including 25 on maxillary prostheses and 8 on mandibular prostheses. The use of digital workflows was limited to one or two steps of the fabrication of the prostheses, and only four studies described a complete digital workflow. The most preferred method for data acquisition was intraoral scanning with or without a cone beam computed tomography combination.

**Conclusion:**

Currently, the fabrication process of maxillofacial prostheses requires combining digital and conventional methods. Simplifying the data acquisition methods and providing user-friendly and affordable software may encourage clinicians to use the digital workflow more frequently for patients requiring maxillofacial prostheses.

## Introduction

Prosthetic rehabilitation of intraoral maxillofacial defects with functionally and aesthetically relevant results is challenging. With advancements in digital technologies, the fabrication methods of intraoral maxillofacial prostheses are constantly emerging and improving [1]. Digital technologies provide adjunct support or sometimes integrate all phases in the fabrication of intraoral maxillofacial prostheses. To the authors’ knowledge, no reliable set of protocols for fabricating intraoral maxillofacial prostheses with a complete digital workflow exists in the literature. Different techniques have been evaluated and compared to know the challenges and drawbacks of digital workflows [2, 3]. The first step in the digital workflow begins with data acquisition. Medical imaging techniques like Computed Tomography (CT), Magnetic Resonance Imaging (MRI), or Cone Beam Computed Tomography (CBCT) provide a three-dimensional volumetric dataset. The acquired CT, MRI, or CBCT data are processed into Data Imaging and Communication in Medicine (DICOM) format. During the CT or CBCT scan, the patient must keep their mouth wide open to keep the tongue and palate apart for an isolated image of the defect [2, 4, 5]. The CBCT can obtain accurate volumetric data, details of the surgical defect, and surrounding tissues. However, it cannot provide soft-tissue details due to the scattering of radiation and low soft-tissue contrast resolution. Therefore, additional data acquisition is required with the help of intra-oral scanners (IOSs) or facial scans, which provide only surface data [1]. IOS provides surface details of oral soft tissue and dentition [6, 7]. IOSs have shown promising results for fabricating single crowns or fixed dental prostheses, either implant or tooth-supported [8], with few clinical reports proving their efficacy in removable prostheses fabrication [9, 10]. Its significance in maxillofacial rehabilitation has remarkably increased over the last 5 years [1]. Previously, digitisation was used for a few steps in the fabrication of maxillofacial prostheses, but with the introduction of new IOSs, complete digitisation is now possible. IOS is the most commonly used method for digital data acquisition. Scanning difficulties in the context of maxillofacial prostheses can pose challenges, particularly when trying to capture defect regions accurately. Defect regions often have irregular shapes and complex geometries; handheld scanners can provide better control and precision in capturing intricate details [11]. Also, a systematic approach could be used to scan smaller sections of the defect region and then stitch the scans together using specialised software. In summary, in the data acquisition step of digital workflow of maxillofacial prostheses, CBCT and CT data are combined with surface data to create a comprehensive model of the defect region. Thereafter, as the second step in the digital workflow, the processed data are converted into standard tessellation language (STL) format and used for designing the prosthesis using different computer-assisted design (CAD) software. Integrating intra-oral scan data with other imaging modalities for a comprehensive representation of the defect region can be challenging. The solution is to utilise specialised CAD software that supports data fusion and integration [12].

The CAD software employs comprehensive tools to sculpt several anatomical details and virtually verify the design of the final prosthesis. The CAD software designed specifically for maxillofacial prosthetics should offer various customisation options to ensure a personalised fit for the defect region. The CAD software is available as open-source or for commercial purposes [4, 12]. The software is technique-sensitive and usually needs the expertise to design the prosthesis digitally. The CAD software can combine and superimpose various data formats like DICOM, STL, and Object, thus providing information about the depth and margins of the area to be rehabilitated. Once the CAD processes have been performed, dental technicians and clinicians can fabricate the subsequent prostheses using Computer Aided Manufacturing (CAM) processes, which is the third step. CAM technologies allows for the creation of highly customised and patient-specific prosthetic devices. The technology enables the fabrication of prostheses that precisely fit the unique anatomical features of an individual patient’s maxillofacial region by using subtractive (milling) or additive technologies like Stereolithography (SLA), Selective Laser Sintering (SLS), Digital Light Processing (DLP), and Fused Deposition Modelling (FDM). Nevertheless, these systems are still evolving for maxillofacial prosthetics, some printers print prosthetic devices with porous structures for reduced weight and support multi-material printing, including biocompatible polymers and resins [13]. Currently, the complete digital workflow is limited to minor and well-defined defects. Considering the increasing number of current publications and the paradigm shift in CAD-CAM, an evaluation of the recent evidence regarding the feasibility of digital workflows in maxillofacial prosthetics is imperative. Therefore, the aim of this systematic review was to assess the current evidence on the feasibility of digital workflow utilised in the fabrication of intraoral maxillofacial prostheses based on the data acquisition methods and the type of software tools used.

## Material and methods

### Study protocol

This systematic review followed Preferred Reporting Items for Systematic Reviews and Meta-Analyses (PRISMA) guidelines. The protocol of this systematic review was framed and registered in the PROSPERO database with registration number CRD42020214217. The study was designed according to the PICO (Population, Intervention, Comparison, Outcome) model:

- ***Population*:** Patients who underwent maxillectomy or mandibulectomy and required an intraoral prosthesis.
- ***Intervention or exposure*:** Maxillary or mandibular prostheses fabricated using digital workflow to rehabilitate the acquired defect.
- ***Comparison:*** No comparison.
- ***Outcome*:** Feasibility and frequency of the digital workflow.

Therefore, the PICO question was: Are fully digitally designed and fabricated prostheses fabrication feasible for rehabilitating maxillectomy and mandibulectomy defects?

### Eligibility criteria

#### Inclusion criteria

- Studies analysing the prosthetic rehabilitation of maxillectomy and/or mandibulectomy defects using digital workflow.
- Studies provide details on the steps for acquiring digital data and the software used to design and fabricate the intraoral maxillofacial prostheses.
- Studies published in English.

#### Exclusion criteria

- Studies analysing orbital, ocular, auricular, nasal, or combination extra-oral prostheses.
- Studies describing the use of maxillary and/or mandibular implant-supported prostheses that are not based on the use of digital workflow.
- Lack of information regarding the data acquisition or software employed using the digital workflow.

### Search strategy and study selection

An electronic search up to November 2023 was conducted in three databases, PubMed, Scopus, and Web of Science (WOS), without applying any additional time or language restriction. The search strategy is shown in Table 1. A subsequent manual search was also carried out in relevant peer-reviewed journals: The Journal of Prosthetic Dentistry, International Journal of Prosthodontics, Journal of Prosthodontics, Journal of Advanced Prosthodontics, and Journal of Indian Prosthodontic Society. The issues of respective journals published through 2010 were screened for any potentially eligible articles. The retrieved articles were imported into a citation manager to discard the duplicates. After removing duplicates, all the articles were screened by two independent reviewers (GS, SKP) based on the relevancy of the title and abstract. The screened articles were then subjected to full-text analysis. Reviewer agreement during the study selection process was estimated using Cohen’s kappa statistics (k-score).

**Table 1:** Systematic search strategy for the focus question.

Focused question	Is fully digitally designed prostheses fabrication feasible for rehabilitating maxillectomy and mandibulectomy defects?	
PICO	Population	Patients who underwent maxillectomy or mandibulectomy and required an intraoral prosthesis.
	Intervention	Maxillary or mandibular prostheses fabricated using digital workflow to rehabilitate the acquired defect.
	Comparison	No Comparison
	Outcome	Feasibility and frequency of the digital workflow.
Search Strategy	(((maxillectomy[Title/Abstract]) OR (mandibulectomy[Title/Abstract])) OR (jaw tumor[Title/Abstract])) AND (((((((intraoral scanner[Title/Abstract]) OR (CAD/CAM[Title/Abstract])) OR (digital impression[Title/Abstract])) OR (CBCT[Title/Abstract])) OR (maxillary obturator[Title/Abstract])) OR (digital workflow[Title/Abstract])) OR (prosthesis[Title/Abstract]))	
Database Search	PubMed, SCOPUS, and Web of Science	

### Data extraction and data items

Two independent reviewers (GS and SKP) conducted the study selection. A third reviewer (NM) was consulted to resolve disagreements at any given point to reach a consensus. The full text of studies fulfilling the inclusion criteria was retrieved and was subjected to data extraction. The following data were extracted from included studies using an Excel spreadsheet (Microsoft, USA): demographic characteristics, year of publication, country, study design, method of data acquisition, software employed, type of prostheses, fabrication method used for prostheses. The retrieved data were subjected to qualitative analysis. The information on the data acquisition process, software, type of prostheses, and fabrication method used for prostheses was tabulated and reviewed to choose the most popular methods.

### Quality assessment of included studies

Two independent reviewers (GS, SKP) performed the quality assessment of the included studies. The Joanna Briggs Institute (JBI) Critical Appraisal Tool for case reports was used to assess the risk of bias in case reports. The tool comprises eight questions; a low risk of bias was considered when ≥ 50% of the answers were ‘yes’, high risk when ≥ 50% were ‘no’, and an uncertain risk of bias if ≥ 50% of the responses were ‘unclear’. The JBI Critical Appraisal Tool for case series comprised 10 questions; the exact method used for case reports was applied for case series while assessing the quality.

## Results

### Study selection

**Figure 1:**
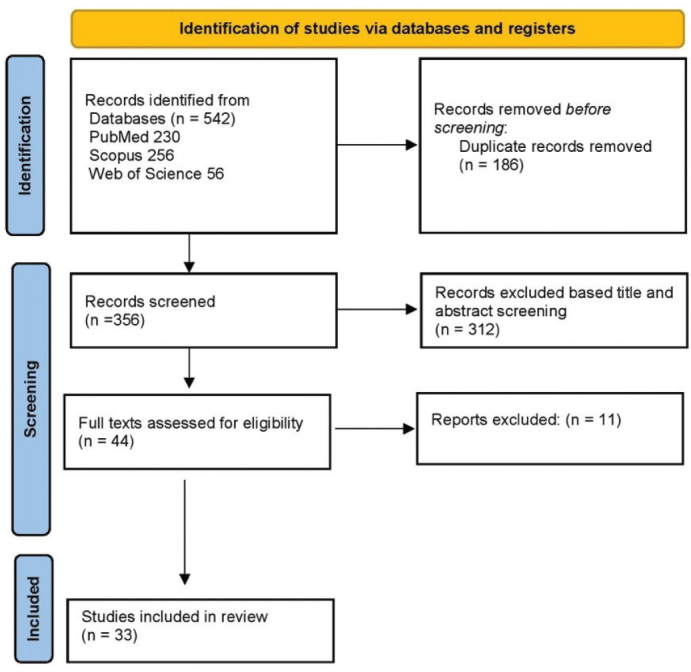
Flowchart of the literature search according to PRISMA guidelines.

A total of 33 articles were included in this systematic review from a pool of 542 articles searched from three databases, namely PubMed/Scopus/WOS (Figure 1). The digital workflow for the intraoral maxillary prosthesis was described in a total of 25 articles which included the case reports, technical notes, case series, and proof of concept; and 8 papers for the mandibular defect rehabilitation which emphasised mainly on digital surgical planning using different software. The data were segregated for the maxillary and mandibular defects and the corresponding prostheses. A meta-analysis could not be performed due to heterogeneous data; most articles were case reports or case series. The reasons for the excluded articles [12–22] are listed in Table 2. The inter-reviewer agreement based on Cohen’s kappa score was 0.82.

**Table 2:** Excluded studies with reasons after full-text evaluation.

Article	Reason for exclusion
Allen et al. 2020 (14)	Inadequate description of means of data acquisition and software tools used
Koyama et al. 2020 (15)	Dental technique; no patient description
de Groot et al. 2020 (16)	The comparison of the reconstructed maxilla with the obturator regarding the quality of life.
Zhang et al. 2020 (11)	Prosthesis fabrication not described
Farook et al. 2020 (12)	*In vitro* study
Revoredo et al. 2018 (17)	Inadequate description of means of data acquisition and software tools used
Weitz et al. 2018 (18)	Prosthesis fabrication not described.
Michenkelis et al. 2017 (19)	Implant-supported maxillary obturator prosthesis
Yoon et al. 2016 (20)	Not described the use of software tools for fabrication of prosthesis
Noh et al. 2016 (21)	Zygomatic implants were used
Elbashti et al. 2016 (22)	*In vitro* study

### Summary and characteristics of the included studies

All 33 included articles [2, 3, 4, 5, 23–51] described the digital workflow for 192 patients, of which 23 were case reports and 10 were case series. The geographic distribution of patient work done had eight from People’s Republic of China [2, 3, 5, 23, 26, 29, 45, 48], five from the United States of America [31, 32, 38, 42, 44], four each from the Republic of Korea [39, 40, 43, 46] and Greece [27, 30, 33, 37], three from the Netherlands [35, 49, 51], two each from Germany [25, 50], Canada [36, 47], and Turkey [24, 34], and one each from Japan [41], Malaysia [4], and Italy [28].

### Maxillary prostheses workflow

For the maxillary prostheses’ fabrication, 25 articles were included, 99 patients were treated, of which 49 were males, 33 were females, and age and gender of 17 patients were not reported. The 25 articles revealed the data acquisition modalities, software employed, prostheses design/types, and fabrication process (Table 3).

**Table 3:** Characteristics of the studies included for maxillary defect.

Author, year	Country	Type of study	No. of patients	Age and gender of the patient	Data acquisition	Software tools used for 3D analysis and integration	CAD Software	Prosthesis type	Fabrication method
Ding et al. 2021(23)	PR China	CR	1	NR	IOS Trios	NR	Dental CAD (3Shape Dental System)	Obturator prosthesis with PEEK framework	3D printed PEEK framework and digital cast
Murat et al. 2021 (24)	Turkey	CR	1	61/F	IOS Trios CBCT	Mimics Materialise	3Matics Materialise	Maxillary obturator and mandibular complete denture	Conventional prosthesis on 3D printed digital cast
Krämer Fernandez et al. 2020 (25)	Germany	CR	1	NR	IOS Trios,	NR	Dental CAD (ExoCad)	3D printed obturator	DLP, 3D Printing
Ye et al. 2020 (26)	PR China	CR	1	NR	Spiral CT, IOS Trios	Mimics Research 22.0; (Materialise) Geomagic Studio	Dental CAD (3Shape Dental System)	1 Obturator with PEEK, 1 PLA obturator	Milled PEEK, FDM, 3D Printing
Tasopoulos et al. 2020 (27)	Greece	CR	1	47/F	CBCT & multi-slice spiral CT and IOS	Blue Sky Plan,	Dental CAD (3Shape Dental System) Meshmixer (Autodesk)	Hollow bulb maxillary obturator	Milled PEEK, Cast fabricated by 3D Printing
Farook et al. 2020 (4)	Malaysia	CR	1	NR	CBCT	Open-source software MITK workbench	Meshmixer Autodesk	Maxillary obturator	3D printed digital cast
Brucoli et al. 2020 (28)	Italy	CS	28	Age range 31–75, M-20 F-8	IOS (TRIOS)	NR	NR	Maxillary obturator	Conventional prosthesis on 3D printed digital cast
Wang et al. 2019 (29)	PR China	CS	10	NR	CBCT Model Scanner	AccuNavi-A2.0,	3Matic Materialise	Maxillary obturator	Conventional prosthesis on 3D printed digital cast
Tasopoulos et al. 2019 (30)	Greece	CR	1	63/M	CT data	Blue Sky Plan software,	Meshmixer Autodesk	Obturator bulb with a complete denture	SLA 3D printer used to fabricate anatomic cast
Soltanzadeh et al. 2019 (31)	USA	CR	1	17/M	Desktop scanner	NR	Dental CAD (3Shape Dental System)	Maxillary cast partial obturator	The framework was 3D printed in Co-Cr alloy using SLS
Palin et al. 2019 (32)	USA	CR	1	55/F	CBCT	Mimics, Materialise;	CAD software (Space Claim)	Silicone definitive prosthesis	3D-printed definitive cast
Michelinakis et al. 2018 (33)	Greece	CR	1	39/M	IOS Trios	Dental Wings Productivity Package software	NR	Cast partial obturator	3D printed resin models, metal framework with SLM technique, The Obturator part milled PEEK.
Murat et al. 2018 (34)	Turkey	CR	1	NR	IOS and CBCT	Mimics Materialise	3 Matics Materialise	Maxillary obturator	Conventional technique on SLA 3D printed resin mould
Kortes et al. 2018(35)	Netherlands	CR	1	46/F	CT and MRI	Synthes ProPlan CMF 3.0, Materialise,	3 Matic, Materialise	Surgical obturator in translucent resin	Direct 3D printed surgical obturator using SLA printer
Bartellas et al. 2018 (36)	Canada	CR	1	NR	CT data	OsiriX Lite Meshmixer	NR	3D printed obturator	3D Printing in FDM using PLA
Ye et al. 2017 (2)	PR China	CS	12	Age range 28–72, M-3 F-9	CT and IOS	Mimics Research 17.0; Materialise	NR	Maxillary obturator	Conventional prosthesis on 3D printed digital cast
Tasopoulos et al. 2017 (37)	Greece	CR	1	65/M	CT data	SimPlant (Materialise) Mimics, Materialise	NR	Maxillary obturator	3D printed digital cast
Rodney & Chicchon 2017 (38)	USA	CR	1	NR	CT data	Mimics, Materialise	NR	Maxillary obturator	Conventionally fabricated on 3D-printed cast
Kim et al. 2017 (39)	Republic of Korea	CR	1	64/F	IOS (TRIOS; 3Shape)	NR	Dental CAD (Exocad)	Complete denture	Conventional prosthesis on 3D printed digital cast
Park et al. 2017 (40)	Republic of Korea	CR	1	59/M	IOS Trios	Geomagic software	LAP tools software	Cast partial obturator	Conventional prosthesis on 3D printed digital cast
Elbashti et al. 2016 (41)	Japan	CR	1	89/F	CBCT scan of the patient’s previous obturator	Mimics 11.11 Materialise	NR	Maxillary obturator	FDM 3D printing using PLA material
Londano et al. 2015 (42)	USA	CR	1	41/M	IOS (Lava)	NR	NR	Cast partial obturator	3D printed digital cast
Jiao et al. 2014 (5)	PR China	CS	11	Age range 25–68, M- 7 F-4	Spiral CT scan	NR	Geomagic software	Cast partial obturator	Resin-positive mould fabricated using SLA
Kim et al. 2014 (43)	Republic of Korea	CR	1	40/F	Master cast scanned using a model scanner	NR	NR	Cast partial obturator	Digital designing of major connector
Jiang et al. 2014 (3)	PR China	CS	18	Age range 42–70, M-13 F-5	3D spiral CT of the maxilla	Mimics (Materialise)	Objet Studio software	Maxillary obturator	Prosthesis fabricated on digital casts

CR: Case Report; CS: Case Series; IOS: Intra Oral Scanner; PEEK: Polyether Ether Ketone; PLA: Poly Lactic Acid; CAD: Computer Assisted Designing; DLP: Digital light processing; CT: Computed Tomography; CBCT: Cone Beam Computed Tomography; RP: Rapid Prototyping; FDM: Fused Deposition Modelling; SLA: Stereolithography; SLM: Selective Laser Melting; NR: Not Reported; M: Male; F: Female.

#### Data acquisition modalities

For the maxillary prostheses’ workflow, data acquisition was done after surgery in all of the cases; the IOS alone was used most frequently [23, 25, 28, 33, 39, 40, 42] followed by CT alone [3, 5, 30, 36–38], CBCT alone [4, 29, 32], CT with an intraoral scanner [2, 26], CBCT with an intraoral scanner [24, 27, 34], and CT with MRI [35]. Trios 3 was the most common IOS used in 11 studies [2, 23–28, 33, 34, 39, 40].

#### Software employed

The STL file format is commonly employed for 3D Printing and CAD. The acquired CT data (DICOM file) was converted to STL file using various commercially available software tools like Mimics [3, 26, 32, 37, 38, 41], Simplant [37], CMF Pro Plan [35], and Geomagic Studio [5, 26, 40]. Additionally, open-source software like Blue Sky Plan software [27, 30], SpaceClaim [32], Dental Wings productivity package [33], and AccuNavi-A [29], were utilised. This comprehensive approach allows professionals to choose the software that best fits their needs and preferences, whether through commercial solutions tailored for specific dental applications or open-source tools that provide flexibility and customisation options. Although, there is no study setup that compares different software to convert DICOM file to STL.

#### Prostheses design

The design of maxillary obturator prostheses involves creating customised, patient-specific devices to address issues such as palatal defects, often resulting from surgical interventions or congenital conditions. CAD software programmes typically facilitate this design process. The included studies mention some specific software tools, 3Matic by Materialise [24, 29, 34, 35], ExoCAD [25, 39], 3Shape design studio software [23, 26, 27, 31], and Geomagic Studio by 3D systems [5, 40]. 3Matic provides tools for manipulating and refining 3D models based on medical imaging data. It may be used to precisely tailor the shape and dimensions of the prosthesis to ensure proper palate coverage and a comfortable fit. Geomagic Studio, part of the 3D Systems software suite, is focussed on processing and manipulating 3D scan data. This software can create accurate 3D models that are the foundation for designing the obturator prosthesis for the patient’s unique oral anatomy. ExoCAD [25, 39] supports rapid prototyping, allowing for quick iterations and adjustments to the prosthetic design. 3Shape Design Studio software [23, 26] shows integration with advanced scanning technologies, anatomic precision, material versatility, and collaboration features, making it an asset in designing patient-specific maxillofacial prostheses. Meshmixer (Autodesk) [4, 27, 30, 36], an open-source software, can be integrated into the digital workflow alongside other CAD software and imaging tools commonly used in maxillofacial prosthetics.

#### Prostheses fabrication

In studies discussing maxillary obturators, the software is commonly utilised to generate a positive mould through 3D printing. The designed digital model is translated into a physical form using 3D printing technology. The 3D printer constructs a digital cast, essentially a tangible representation of the maxillary anatomy. Subsequently, the prosthesis is manufactured using conventional methods. Most of the included studies utilised this method of prostheses fabrication [2, 3, 24, 28, 29, 34, 38–40, 42]. Once the positive mould is obtained through 3D printing, conventional methods may include casting, milling, or 3D printing depending on the materials used for the final prosthesis. Common materials include acrylics or other biocompatible materials like polyether ether ketone (PEEK) and polylactic acid (PLA) which are suitable for oral prosthetics.

### Mandibular prostheses workflow

Eight articles on rehabilitating mandibular defects comprised 93 patients, 51 males and 30 females, and gender of 12 patients was not reported (Table 4). There was no report of the direct digital workflow involved in the prosthetic restoration of mandibular defects. Rehabilitation for mandibular resection cases comprises reconstruction with vascularised osseous free flap, mostly fibula or the iliac crest, followed by the implant-supported prosthesis [52]. The reconstructive surgery was done with the help of digital surgical planning, which comprises a scan of the fibula and the mandible, and the fabrication of surgical resection guides using 3D printed technology. The digital surgical planning resulted in proper contouring of the mandible, thus resulting in the appropriate fit of the prosthesis and indirectly improving the quality of prosthetic rehabilitation [46, 49].

**Table 4:** Characteristics of the studies included for mandibular defects.

Author year	Country	Type of study	No. of patients	Age (in years) and gender of the patient	Data acquisition	Software tools used for 3D analysis and integration	CAD software	Prosthesis type	Fabrication method
Williams et al. 2020 (44)	USA	CS	12	NR	CBCT, Fibula CT, IOS	Blue Sky plan software,	Meshmixer Autodesk	Implant retained fixed prosthesis	3D Printing using an SLA printer
Ren et al. 2018 (45)	PR China	CS	30, 15 in the CAS group and 15 in the conventional group	Age range 21–63, M-19 F-11	CT scans of the mandible CT scan of lower extremity	Mimics 16.0 (Materialise)	NR	Implant-supported prosthesis	Cutting guides and a 3D model made in polyamide using a 3D printer
Oh et al. 2018 (46)	Republic of Korea	CR	1	44/F	CBCT	Mimics 16.0 (Materialise)	Dental design software	Hybrid monolithic zirconia prosthesis	3D printed resin guide for implant placement
Chuka et al. 2017 (47)	Canada	CS	19, 8 with SDS 11 without SDS	Mean Age 47, M-11 F-8	NR	Surgical Design and Simulation Plan	NR	NR	3D printed anatomical models, surgical cutting and drilling guides, and positioners.
Zhang et al. 2016 (48)	PR China	CS	22, 8 CAS 14 conventional	Age range 16–52, M-14 F-8	CT scan of the maxillofacial region and lower extremities	Surgicase 5.0 software (Materialise),	Geomagic Studio software.	Removable Dental Prostheses	Mandibular stereomodel, mandibular and fibula cutting guides, and mandibular reconstructive guides
Schepers et al. 2015 (49)	Netherlands	CS	7	Age range 43–84, M-5 F-2	CBCT of the mandible, CT of legs	Proplan CMF, Simplant	Geomagic	Fixed temporary implant-supported prosthesis	Acrylic prostheses
Freudlsperger et al. 2014 (50)	Germany	CR	1	56/M	CT, Angio CT and MRI of the fibula	Surgicase (Materialise)	Simplant (Materialise)	Implant-supported prosthesis	An SLA mandibular model and a titanium bar was prefabricated on it.
Schepers et al. 2013 (51)	Netherlands	CR	1	54/M	CBCT, Fibula CT, Intraoral scanner (Lava)	Surgicase CMF software (Materialise)	Simplant Crystal (Materialise)	Implant-supported prosthesis	Intermediate occlusal guide was 3D printed. Milled titanium framework.

CR: Case Report; CS: Case Series; CAS: Computer Assisted Surgery; SDS: Surgical Design and Simulation; CT: Computed Tomography; CBCT: Cone Beam Computed Tomography; SLA: Stereolithography; NR: Not Reported; CAD-CAM: Computer Assisted Designing/Computer Assisted Manufacturing; M: Male; F: Female.

#### Software employed

The software used for the data acquisition in mandibular reconstruction cases were Surgicase CMF [48, 49, 51], Mimics [45, 46], Proplan CMF [49], and Blue-Sky Plan [44]. The software provides the DICOM data, which allows the creation of virtual models of the maxillofacial region and the fibula. This, in turn, facilitates the further simulation of mandibular reconstructive surgery. The software allows surgeons to plan and simulate complex surgeries using 3D imaging data. It enables the creation of patient-specific anatomical models, surgical guides, and implants. This personalised approach helps surgeons visualise the patient’s anatomy in three dimensions and plan the surgery more accurately.

#### Prostheses design

Meshmixer (Autodesk) [44], Geomagic [48, 49], and Simplant (Materialise) [49–51] software were used for designing prosthesis, which were implant supported in most of the mandibular cases [45, 49–51].

#### Prostheses fabrication

3D Printing was used as the common modality to fabricate the prostheses [44, 46, 47], although the framework was sometimes milled using titanium [50, 51].

### Quality assessment data

Most of the case reports included in this review showed a low risk of bias according to the JBI Critical Appraisal Tool [53], except seven studies [4, 23, 25, 26, 34, 36, 38] showed a high risk of bias. (Table 5) The high risk of bias was attributed to the fact that the studies did not describe the patient’s demographic and history clearly; some even did not explain the post-intervention clinical condition. For the Case series (Table 6), five studies showed low risk [2, 3, 5, 45, 47], and four presented unclear risk [28, 29, 48, 49], and one study showed high risk of bias [44]. The unclear risk is mainly attributed to inappropriate statistical analysis and when the studies do not have consecutive inclusion of participants. The high risk was when there were no clear criteria for inclusion in the study, and the clinical condition was not reported aptly.

**Table 5:** Quality assessment of case reports using the Joanna Briggs Institute critical appraisal tools.

Author year	Were the patient’s demographic characteristics clearly described?	Was the patient’s history clearly described and presented as a timeline?	Was the current clinical condition of the patient on presentation clearly described?	Were diagnostic tests or assessment methods and the results clearly described?	Was the intervention or treatment procedure clearly described?	Was the post-intervention clinical condition clearly described?	Were adverse events (harms) or unanticipated events identified and described?	Does the case report provide takeaway lessons?	Risk of bias
Ding et al. 2021(23)	N	N	N	N	Y	U	N	Y	High
Murat et al. 2021(24)	Y	Y	Y	Y	Y	Y	Y	Y	Low
Krämer Fernandez et al. 2020 (25)	N	N	N	N	Y	U	N	Y	High
Ye et al. 2020 (26)	N	N	N	N	Y	U	N	Y	High
Tasopoulos et al. 2020 (27)	Y	Y	Y	Y	Y	Y	N	Y	Low
Farook et al. 2020 (4)	N	N	N	N	Y	N	N	Y	High
Tasopoulos et al. 2019 (30)	Y	Y	Y	Y	Y	Y	Y	Y	Low
Soltanzadeh et al. 2019 (31)	Y	Y	Y	Y	Y	Y	N	Y	Low
Palin et al. 2019 (32)	Y	Y	Y	Y	Y	Y	Y	Y	Low
Michelinakis et al. 2018 (33)	Y	Y	Y	Y	Y	N	Y	Y	Low
Murat et al. 2018 (34)	N	N	N	N	Y	N	N	Y	High
Kortes et al. 2018 (35)	Y	Y	Y	Y	Y	Y	N	Y	Low
Bartellas et al. 2018 (36)	N	N	N	N	Y	N	Y	Y	High
Tasopoulos et al. 2017 (37)	Y	Y	Y	Y	Y	Y	N	Y	Low
Rodney& Chicchon 2017 (38)	N	N	N	N	Y	N	N	Y	High
Kim et al. 2017 (39)	Y	Y	Y	Y	Y	Y	Y	Y	Low
Park et al. 2017 (40)	Y	Y	Y	Y	Y	Y	N	Y	Low
Elbashti et al. 2016 (41)	Y	Y	Y	Y	Y	Y	N	Y	Low
Londano et al. 2015 (42)	Y	Y	Y	Y	Y	Y	N	Y	Low
Kim et al. 2014 (43)	Y	Y	Y	Y	Y	Y	Y	Y	Low
Oh et al. 2018 (46)	Y	Y	Y	Y	Y	Y	N	Y	Low
Freudlsperger et al. 2014 (50)	Y	Y	Y	Y	Y	Y	N	Y	Low
Schepers et al. 2013 (51)	Y	Y	Y	Y	Y	Y	N	Y	Low

Y: Yes; N: No; U: Unclear; NA: Not applicable.

**Table 6:** Quality assessment of case series using the Joanna Briggs Institute critical appraisal tools.

Author year	Were there clear criteria for inclusion in the case series?	Was the condition measured in a standard, reliable way for all participants in the case series?	Were valid methods used to identify the condition for all participants included in the case series?	Did the case series have consecutive inclusion of participants?	Did the case series have a complete inclusion of participants?	Was there clear reporting of the demographics of the participants in the study?	Was there clear reporting of clinical information of the participants?	Were the outcomes or follow-up results of cases reported?	Was there clear reporting of the presenting site(s)/clinic(s) demographic information?	Was statistical analysis appropriate?	Risk of Bias
Brucoli et al. 2020 (28)	Y	Y	Y	Y	Y	Y	Y	Y	N	N	Unclear
Wang et al. 2019 (29)	Y	Y	Y	Y	Y	N	N	Y	Y	Y	Unclear
Ye et al. 2017 (2)	Y	Y	Y	Y	Y	Y	Y	Y	Y	Y	Low
Jiao et al. 2014 (5)	Y	Y	Y	Y	Y	Y	Y	Y	Y	N	Low
Jiang et al. 2014 (3)	Y	Y	Y	Y	Y	Y	Y	Y	Y	Y	Low
Williams et al. 2020 (44)	N	U	U	N	Y	N	N	Y	U	N	High
Ren et al. 2018 (45)	Y	Y	Y	Y	Y	Y	Y	Y	Y	Y	Low
Chuka et al. 2017 (47)	Y	Y	Y	Y	Y	Y	Y	Y	Y	Y	Low
Zhang et al. 2016 (48)	Y	Y	Y	N	Y	Y	Y	Y	N	Y	Unclear
Schepers et al. 2015 (49)	U	Y	Y	N	Y	Y	Y	Y	N	Y	Unclear

Y: Yes; N: No; U: Unclear; NA: Not applicable.

## Discussion

Prosthetic rehabilitation of ablative defects remains a clinical challenge due to the inherent characteristics of the maxillofacial patient. In this sense, implementing digital technologies in this field can provide potential benefits when rehabilitating these patients. Fully digital workflows are still in the nascent stage for maxillary and mandibular intraoral prostheses. Initially, the trend was to capture the digital image, and the most frequent method was to print the definitive cast with the 3D printing technique and then fabricate the prosthesis with conventional methods [34, 38–40]. The clinical workflow still requires conventional prosthesis fabrication methods like fabricating the metallic framework with lost wax technique, wax-up and analysation, including some digital steps. The currently available sources simplify the data acquisition in combination with affordable software to design and fabricate maxillofacial prosthetics.

The included studies reported that IOS alone was the most frequently used digital data acquisition technique, producing possible results from the present systematic review [23, 25, 28, 33, 39, 40, 42]. However, the most predictable results were generated when IOS was used in combination with the CT or CBCT to generate the 3D digital casts for the maxillary defects, with all the anatomical details recorded for the fabrication of the maxillary prosthesis [2]. Prostheses fabricated with conventional techniques on the digital models presented good clinical efficacy, thus signifying that the digital casts are adequate for clinical usage [2]. In an *in vitro* study conducted by Elbashti et al. [54], CBCT and the IOS data were used to evaluate the feasibility and accuracy of digitising the edentulous maxillectomy cast and compared it with the conventional technique. It proved feasible with certain limitations like the exact simulation of the oral environment, for example saliva and soft tissue. The use of IOS in maxillofacial prostheses has gained popularity in the last decade and has become an alternative to conventional impression-making [1]. Ye et al. [26], Tasopoulos et al. [27], Kramer et al. [25], and Michelinakis et al. [33] have reported the fully digital workflows for intraoral maxillofacial prostheses involving all the fabrication steps.

Cast Partial Obturator was given in 16 cases [5, 31, 33, 40, 42, 43] out of 99 included maxillary defect cases. CAD-CAM technologies can be effectively applied in the design and fabrication of the Removable Partial Denture frameworks, offering several benefits, such as automatic determination of the insertion path and digital surveying, eliminating unfavourable undercuts, and reducing fabrication time [10, 55]. The same principles could be applied to maxillofacial prosthesis fabrication, which reduces unfavourable undercuts and a proper insertion path. The most used commercially available software was Mimics (Materialise), as seen in the included studies [3, 26, 32, 37, 38, 41, 45, 46], and the most common open-source software was Meshmixer (Autodesk) [4, 27, 30, 36], The commonly used prosthesis design software is Geomagic Studio software [5, 26, 40, 48, 49], The advantage of dental CAD software is that different files, such as STL, DICOM, and OBJ, could be superimposed, and valuable information could be generated about the area to be rehabilitated. Dental professionals and prosthodontists can simplify the design process of maxillary obturator prostheses by using CAD software programmes like 3Matic, ExoCAD and 3Shape Design Studio software. These tools enable the creation of virtual models that guide the fabrication of personalised prosthetic devices, contributing to better patient outcomes and improved comfort and functionality for individuals with maxillary defects.

Kortes [35] reported the fabrication of a hollow surgical obturator using CT and MRI data. The digital design permits a hollow obturator, reducing the prosthesis’s weight. Tasopoulas et al. [27] attempted the same in their case by acquiring the scan using IOS and CT, digitally designing the obturator framework, and milling the prosthesis using modified PEEK material, resulting in a highly biocompatible, lightweight prosthesis.

By combining the precision of CAD software and the versatility of 3D printing technology, clinicians can achieve a highly accurate and patient-specific positive mould, which is the foundation for the subsequent conventional fabrication of the maxillary obturator prosthesis. This approach allows for a more tailored and efficient manufacturing process, improving fit, comfort, and overall patient satisfaction. The materials suitable for maxillofacial prosthetics include biocompatible resins and polymers like PEEK and PLA. For the substantial use of complete digital workflow in the maxillofacial prosthesis, more research is required on material compatibility with 3D printing materials.

Four [44, 45, 49, 51] of the eight included mandibulectomy studies reported rehabilitation with implant-supported prostheses. The position of the dental implants to support maxillofacial prosthesis should be virtually planned. Digital surgical planning evaluates the bone plate relationship for positioning of patient-specific dental implants, thus providing aesthetic and functional prosthetic solutions and restoring correct occlusion [49]. The CAD-CAM techniques for mandibular reconstruction offer new vistas for the digitalised planning of reconstructive surgery, which results in aesthetic outcomes and prosthetic rehabilitation [56]. It improves functional outcomes due to accurate postoperative maxillomandibular relationships [45].

Certain limitations of this systematic review were that most of the included studies were not clinical trials but clinical reports or case series that provided inadequate evidence. Many included studies showed unclear or high risk of bias, which denoted no clear criteria for inclusion in the study, and the clinical condition was not reported aptly; moreover, the patient’s demography and post-intervention clinical condition were unclear. These factors should be kept in mind when designing future studies. Many studies have not described the detailed use of software tools, techniques, and materials to fabricate maxillofacial prostheses, which is essential in developing a reliable set of protocols for the digital workflow. The detailed description in future studies will help formulate a digital workflow which will reduce bias.

Furthermore, the authors did not find any implant-supported obturators based on digital workflow either with dental or zygomatic implants. Currently the evidence is limited to defect anatomical data acquisition but not the implant data acquisition [19, 21]. Further studies are recommended in order to evaluate the feasibility of digital workflow for any implant-supported obturators prostheses.

The absence of randomised clinical trials may be attributed to the recent advancements in systems for the digital workflow for maxillofacial prosthetic rehabilitation. The software is often expensive, and many dental professionals lack proficiency in CAD software. With the increasing demand for digital workflows in maxillofacial rehabilitation, biomechanical engineers or software designers need to develop more user-friendly and affordable software that is accessible to dental professionals. The geographic distribution shows that the included studies were limited to a few countries. There is a need to broaden the scope and usage of the digital workflow in various geographic locations worldwide.

## Conclusion

Despite the limited evidence, it can be concluded that the use of digital workflows was restricted to one or two steps and not all in the fabrication of the intraoral maxillofacial prostheses. The fabrication process of maxillofacial prostheses usually involves combining digital and conventional methods. Simplifying the data acquisition methods and providing user-friendly and affordable software will encourage clinicians to use the digital workflow more frequently for patients requiring maxillofacial prostheses. Further studies are needed to standardise the steps of digital workflow for maxillofacial rehabilitation.
